# Read Medical Journals

**Published:** 1887-11

**Authors:** 


### Read Medical Journals.

Dr. T. L. Brown writes as follows to the *Medical Advance*, August, 1887: I secured a very important case, many years ago, and through this one case a number of others were brought to me. I never knew until months afterwards how I happened to be selected. It was in this way: One night, at quite a late hour, I was called to see the family of a prominent New Hampshire official, temporarily staying in our town, to whom I was a perfect stranger. After I had discharged myself, and quite a while afterwards, I learned that as soon as the gentleman found that he required a physician, instead of asking the landlord of his hotel, or appealing at some drug store for the name of a doctor, he took a carriage and drove to the house of the postmaster. "I want a doctor," said he. "Tell me which one of the doctors of this city takes the largest number of journals?" The postmaster referred him to me. As the gentleman was leaving the house he said to the postmaster: "A man who takes the journals of his profession is well read and up with the time, and that is the doctor I want to treat me and my family."

